# Analyzing the public discourse on works of fiction – Detection and visualization of emotion in online coverage about HBO’s Game of Thrones

**DOI:** 10.1016/j.ipm.2015.02.003

**Published:** 2016-01

**Authors:** Arno Scharl, Alexander Hubmann-Haidvogel, Alistair Jones, Daniel Fischl, Ruslan Kamolov, Albert Weichselbraun, Walter Rafelsberger

**Affiliations:** a Department of New Media Technology, MODUL University Vienna, Am Kahlenberg 1, 1190 Vienna, Austria; b WebLyzard Technology, Puechlgasse 2/44, 1190 Vienna, Austria; c University of Applied Sciences HTW Chur, Faculty of Information Sciences, Pulvermuehlestrasse 57, CH-7004, Chur, Switzerland

**Keywords:** Knowledge extraction, Web intelligence, Visual analytics, Interactive dashboard, Television series, Game of Thrones

## Abstract

This paper presents a Web intelligence portal that captures and aggregates news and social media coverage about “Game of Thrones”, an American drama television series created for the HBO television network based on George R.R. Martin’s series of fantasy novels. The system collects content from the Web sites of Anglo-American news media as well as from four social media platforms: Twitter, Facebook, Google+ and YouTube. An interactive dashboard with trend charts and synchronized visual analytics components not only shows how often Game of Thrones events and characters are being mentioned by journalists and viewers, but also provides a real-time account of concepts that are being associated with the unfolding storyline and each new episode. Positive or negative sentiment is computed automatically, which sheds light on the perception of actors and new plot elements.

## Highlights

• “Westeros Sentinel” – a visual analytics dashboard for Game of Thrones.
• Extraction of affective and factual knowledge from news and social media coverage.
• Emotional categories from semantic knowledge bases.
• Automated annotation services for contextualized information spaces.
• Interactive visualizations to explore context features.

## 1 Introduction

Television as a medium is currently undergoing significant systematic changes. A decade ago the experience of watching a television program typically involved one device (television) in one location (home), from a single source (station) and at a scheduled time (broadcast). Now the experience has become much more heterogeneous, with the introduction of a wide range of multimedia devices (PCs, smartphones, tablets, gaming consoles) and Internet video streaming services allowing users to consume old and new content at any time, at any place (Narasimhan and Vasudevan, 2012, Zeadally et al., 2011). Television has been an important sector of the entertainment industry since its invention, but this paradigm shift has caused a breakdown in the traditional models and methods of measuring viewership – an important metric when selling advertising time and generating revenue.

At the same time, new media technologies have added a new take on the social component of the television watching experience (Napoli, 2013). With these technologies comes a wealth of platforms, services and applications where not only audiences, but critics, actors, producers, and marketers alike can share their experiences, discuss important events, express views and provide interpretations. The very idea of measuring viewership is becoming less important. In its place, the notion of audience engagement has come to dominate contemporary thinking about the impact and reach of particular television programs (Napoli, 2013).

Wakamiya, Lee, and Sumiya (2011) and Napoli (2013) discuss limitations of traditional methods such as those used by Nielsen Media Research (www.nielson.com), the current industry-wide standard for audience ratings. The authors demonstrate the advantages of performing a complementary analysis of social network activity. Such novel approaches have triggered Nielsen’s recent acquisition of SocialGuide, a Web site for the analysis of audience engagement around TV programs (www.nielsensocial.com). In a partnership with Twitter, they release weekly *Top Ten Nielsen Twitter Ratings* for television programs – indicating that the industry is fully aware of the changing media landscape.

These approaches focus on only a small portion social activity and only measure audience engagement, without trying to fully understand the factors triggering and influencing public discourse. The webLyzard Web intelligence platform (Scharl, Hubmann-Haidvogel, Sabou, Weichselbraun, & Lang, 2013) provides a more comprehensive portfolio of analytical methods. Its visual dashboard supports different types of information seeking behavior such as browsing, search, trend monitoring and visual analytics. The context-sensitive dashboard goes beyond charts and similar statistical representations. Using multiple coordinated view technology and real-time synchronization mechanisms (see Section 2.3), it helps to analyze and organize the extracted knowledge, and to navigate the information space along multiple dimensions.

Due to the massive amount of expressive, creative and subjective data from these sources, our approach is grounded in content-aware semantic processing and sentiment analysis (Weichselbraun, Gindl, & Scharl, 2013) to help analysts make sense of factual and affective knowledge in the data. We use SenticNet 3 as a language resource for concept-level sentiment analysis to unveil the perception of the storyline or characters of a particular program (Cambria, Olsher, & Rajagopal, 2014). Its affective knowledge space is wrapped around a four-axis structure, assigning terms to the categories *pleasantness, aptitude, attention*, and *sensitivity*. The extensiveness of SenticNet 3 with approximately 30,000 terms guarantees high coverage and ensures the discovery of all relevant concepts. A concept-level approach also facilitates the visualization of observed patterns, and plays a crucial role in the visual analytics dashboard, which first grants users an overview of the media activity around a television program and then allows them to search, filter and sort using similarity metrics and sentiment cues. The resulting system provides analysts and researchers with a comprehensive corpus based on online media coverage, and with advanced text mining tools that allow an unprecedented level of transparency about emerging trends and the impact of specific events on the public discourse.

We have chosen the popular HBO television program *Game of Thrones* as a case study, because the series has established a reputation for being actively discussed via social media channels (Hernandez, 2013, John, 2014). Our proposed solution, the *Westeros Sentinel*, is an application built on top of the webLyzard platform. It processes online sources in real time and extracts actionable knowledge from these sources. The system’s integrated information space allows for a social construction of meaning by journalists, film critics, viewers and readers of the novels, as well official HBO announcements and the postings of members of the Games of Thrones cast.

The remainder of this paper is structured as follows: Section 2 introduces the Web intelligence platform that the *Westeros Sentinel* is built on, including an overview of the data acquisition and knowledge extraction services to continuously update the portal. The dashboard description of Section 3 presents a high-level overview of the various elements of the information exploration and retrieval interface. Section 4 then describes visual means to analyze affective knowledge contained in the gathered data, including an interactive *word tree* that captures the lexical context of search queries (Section 4.1), a *radar chart* to track emotional categories (Section 4.2), and an *entity map* to identify relations among (Section 4.3). Section 5 concludes the paper and discusses promising areas for follow-up research.

## 2 Technology platform

Media monitoring systems have been designed for analyzing social media streams across various domains including sports (Marcus et al., 2011), politics (Diakopoulos et al., 2010, Shamma et al., 2009, Shamma et al., 2010) and climate change (Hubmann-Haidvogel, Scharl, & Weichselbraun, 2009), focusing on specific aspects like (sub-)event detection (Adams, Phung, & Venkatesh, 2011), classification (Hubmann-Haidvogel et al., 2009) and the analysis of video broadcasts (Diakopoulos et al., 2010). Such media monitoring tools face two major challenges: (i) collect, analyze and structure very large document collections originating from sources that are heterogeneous in terms of their authorship, formatting, style (e.g., news article versus tweets) and update frequency (weekly, daily or real-time); (ii) provide an interactive interface to select a relevant subset of the information space, and to analyze and manipulate the extracted data.

To process and enrich streams of information from unstructured, structured and social evidence sources, the *Westeros Sentinel* utilizes the webLyzard Web intelligence platform (www.weblyzard.com), whose Web crawling and text processing components have been designed specifically for scalability, modularity, and adaptability. webLyzard draws upon expertise from many disciplines including human–computer interaction, information visualization, text mining, and natural language processing - in order to shed light on the perceptions of different stakeholders, reveal flows of relevant information, and provide indicators for assessing the effectiveness of public outreach activities.

### 2.1 Content acquisition and preprocessing

Real-time content acquisition services are crucial for tracking the public discourse surrounding popular TV series and other media events with a significant number of followers. The resulting corpus contains diverse opinions of globally distributed individuals with very heterogeneous backgrounds. The *Westeros Sentinel* harvests documents from 150 English-language news media sites (US, CA, UK, AU, NZ) and social media platforms including Twitter, Facebook, Google+ and YouTube.

Handling the abundance and dynamics of news and social media content requires efficient pre-processing to eliminate irrelevant content at an early stage. This considerably reduces the number of documents to be processed by more advanced and therefore resource-intensive components. The *Westeros Sentinel* applies a domain specificity measure which combines and weighs different black- and whitelists to assess the relevance of a document. Table 1 presents a selection of filter expressions used for identifying Game of Thrones characters based on the descriptions of “A Wiki of Ice and Fire” (awoiaf.westeros.org), a fan-built site that provides comprehensive information on the television show and the corresponding books.

### 2.2 Factual and affective knowledge extraction

A continuously evolving knowledge repository gathered from online sources helps to better understand social networks and the dynamic relations among their participants. Which fictional character has the most negative reputation among social media users? Who are the most visible actors among the cast members of the current episode, and what are mainstream media associating with them? The platform’s seamless integration of factual (concepts, instances, relations) and affective (sentiment, opinions) knowledge helps to answer such questions.

• *Factual Knowledge*: The *Westeros Sentinel* portal applies *Recognyze* (Weichselbraun, Streiff, & Scharl, 2014), a named entity recognition and resolution component which uses external knowledge sources such as DBpedia, Freebase and GeoNames to identify locations, persons and organizations in the gathered corpus, and to align these entities with items contained in linked open data archives. The system not only identifies and classifies referenced named entities, but also grounds them to external knowledge items – e.g. to the linked open data entry for the fictional character *Arya Stark* (www.dbpedia.org/page/Arya_Stark).
• *Affective Knowledge:* includes sentiment and other emotions expressed in a document, which are captured and evaluated by opinion mining algorithms. The approach used by the *Westeros Sentinel* system relies on sentiment lexicons, which contain known sentiment terms and their respective sentiment value (Weichselbraun et al., 2014, Weichselbraun et al., 2013). The ratio of positive and negative terms found in the vicinity of a target term is used as an indicator of overall polarity. The accuracy of the knowledge extraction process is improved by considering linguistic features such as negations and intensifiers (Weichselbraun et al., 2013), and by creating a contextualized and domain-specific version of the sentiment lexicon (Weichselbraun, Gindl, et al., 2014). Once *Westeros Sentinel* users query for a named entity, the system identifies associated topics and computes the average sentiment toward this entity for the specified time interval. The visualizations introduced in Section 4 reflect sentiment information by means of color coding, ranging from red (negative) to gray (neutral) and green (positive). Sentiment is shown with variable saturation, depending on the degree of polarity – vivid colors indicate emotional articles or postings, and lower saturation a more factual online coverage.

### 2.3 Exploring contextualized information spaces

As outlined in the introduction, the goals of the *Westeros Sentinel* project go beyond providing a current stream of Game of Thrones-related information from the various online sources listed in Section 2.1. Understanding the reach and impact of expressive media requires extracting prevalent topics in the discussions, and the aggregation and clustering of the opinions voiced by the various parties. This is a complex task that involves (i) analytic methods to extract the knowledge, and (ii) visual tools to convey the knowledge in an intuitive manner:

• The system detects and tracks emerging topics that are frequently mentioned in a given data sample - typically, an archive of Web documents crawled from relevant online sources. Advanced data mining techniques then extract a variety of contextual features from the multidimensional document space.
• A portfolio of synchronized visualizations shows the evolution of the dataset along the dimensions defined by these contextual features (temporal, geographic, semantic, and attitudinal), and provides drill-down functions for in-depth analyses. A key strength of the interface is its use of *multiple coordinated views*, also known as linked or tightly coupled views in the literature (Hubmann-Haidvogel et al., 2009), where a change in one of the views triggers an immediate update of the others (e.g., when a new document is viewed, the maps pan and zoom to the context of this document).

**Fig. 1:**
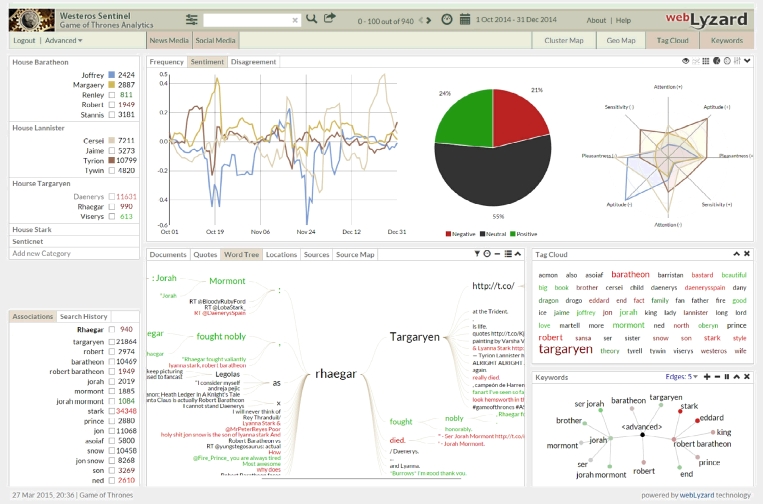
Screenshot of the *Westeros Sentinel* dashboard, showing results for a query on the character “Rhaegar Targaryen” based on online coverage in the fourth quarter of 2014.

The screenshot in Fig. 1 exemplifies this process. The system automatically extracts the dominant issues that are discussed in conjunction with a topic or entity such as the fictional character “Rhaegar Targaryen”. The dashboard displays them through a set of charts that show frequency, sentiment, and observable level of disagreement among sources. At any given time, only a subset of the document space is displayed, depending on the selected source, time interval and affective value – e.g., positive news media articles about an episode published in the first three days after its broadcast. Various visualizations are available to shed light on the semantic context of a topic (see Section 4).

## 3 Dashboard of the *Westeros Sentinel*

**Fig. 2:**
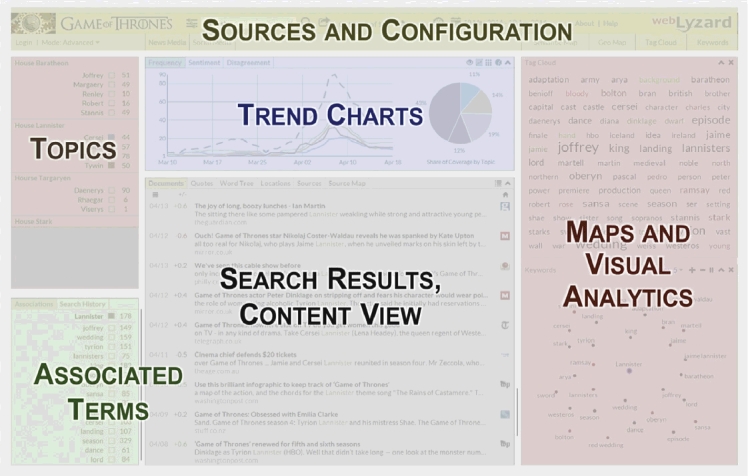
Overview of the *Westeros Sentinel* dashboard.

The information exploration and retrieval interface (=“dashboard”) of the *Westeros Sentinel* helps users to interactively identify, track and analyze coverage about actors or plot elements. The dashboard is divided into six main content areas as shown in Fig. 2 :

1. *Sources and Configuration:* Drop-down elements in the upper menu let users choose a time interval, the document source (news or social media), and the global sentiment filter setting (unfiltered, positive, negative). A search bar allows users to query the corpus of news and social media documents through plain text searches. To the right of the search bar, users can see the currently selected and total number of matches of their queries – for example, “0–50 out of 989” in Fig. 1.
2. *Topics:* The upper left window of the dashboard contains the topic management and content navigation. Users can (a) click on a term to trigger the full text search; (b) use topic markers to select the topics to be shown in the charts; (c) select the first five topics (or deselect all) with the ‘chart’ symbol next to a category name; and (d) add/modify topics and email alerts with the topic editor (‘settings’ symbol).
3. *Trend Charts:* Below the source selection, interactive trend charts show (i) the frequency of selected topics in the specified time interval, (ii) the average sentiment regarding these topics, and (iii) the level of disagreement – i.e., the standard deviation of sentiment which reveals how polarizing the online coverage is.
4. *Search Results and Content View:* The content view shows the search results in seven different tabs: documents, sentences, word tree, entities, entity map, sources, and source map. The first of these tabs is the document view, where results are presented in a table with several interactive controls: (i) mouse-over allows previewing documents; (ii) a first click selects a document and shows its content in extended form next to a yellow asterisk; (iii) a second click switches to full text view, which reveals the document’s annotations including keywords, location, sentiment, and relevance.
5. *Associated Terms:* The lower left view of the dashboard displays a list of terms associated with a selected topic, based on the selected source and time interval.
6. *Maps and Visual Analytics:* Visual means to investigate stakeholder associations with a search term are the keyword graph (hierarchical structure) and the adaptive tag cloud (alphabetical structure). Other visualizations include geographic maps to show the regional distribution of search results, ontology graphs to provide a conceptual overview of the domain, and information landscapes to reveal major document clusters. The maps are synchronized and can be re-positioned using drag-and-drop operations.

### 3.1 Synchronisation mechanism

Following an evolutionary systems development approach (Scharl, 2000), rapid feedback cycles and agile software development (Dönmez & Grote, 2011) have been instrumental in conceptualizing and implementing the dashboard. The synchronization of dashboard components is fundamental to the platform’s ability to provide context-specific recommendations.

To gain insight into the user experience of this synchronization mechanism, usability evaluations were conducted in regular intervals. The aim of these evaluations was to determine strengths and weaknesses of the interaction design, distinguishing two types of assessment: (i) *heuristic evaluation*, where experts examined the interface and judged the extent to which it is compliant with established usability principles; (ii) *formative usability tests*, where users were observed while working on predefined tasks in realistic settings. Their gaze data was recorded with a *Tobii X60 Eye Tracker* to generate heat maps that show which elements of the dashboard were used to complete a given task, and in which sequence.

The feedback showed that test users have little difficulty using the system after a short training session. They appreciate the synchronized views to keep track of the semantic and geospatial context of their search queries. For untrained first-time users, however, the complexity of the dashboard can be overwhelming. Therefore a simplified mobile version of the dashboard has been developed, specifically tailored to the requirements of tablets, smartphones and other mobile devices. Fully compliant with HTML5 and other Web standards, the mobile dashboard behaves like a native app on iPhone/iPad and Android devices. For desktop users, it provides a simple yet powerful alternative to the multiple coordinated views of the regular dashboard.

### 3.2 Maps and visual analytics

The right side of the dashboard offers a suite of visualizations that aggregate information along two main context dimensions: geographic and semantic. The *geographic map* shows the locations of documents based on analyzing their textual content – a process typically referred to as “geo-tagging” (Scharl & Tochtermann, 2007). Such a display, however, is of limited use when targeting fictional cities and kingdoms – unless one would create a map and gazetteer for the *Seven Kingdoms* and the fictional content of *Westeros*. The semantic dimension of the information space is exposed by three different views that leverage increasingly complex semantics:

• the *tag cloud* is derived from the most frequently mentioned keywords in the information space; the color of keywords indicates their sentiment;
• the *information landscape* displays clusters of topically related documents, thus depicting intrinsic semantic relations between documents;
• the *ontology graph* displays an a priori constructed semantic model of the domain and assigns each document to the best-matching concept.

Since these visualizations have already been introduced in previous work (Scharl et al., 2013), the following section focuses on components specifically developed and extended for the *Westeros Sentinel*, taking into account the importance of named entities – i.e., actors and fictional characters, as well as the dynamic and complex set of relations among these entities.

## 4 Visualizing the public discourse about works of fiction

This section describes visualization techniques of the *Westeros Sentinel,* including the *word tree* to represent the lexical context of query terms (Section 4.1), the *radar chart* as a visual tool to depict affective knowledge along emotional categories (Section 4.2), and the *entity map* to summarize the occurrence of named entities in a corpus, including the sentiment toward those entities and the relations among them (Section 4.3).

### 4.1 Lexical context

Once a user has entered a search query, the system ranks the matching results by relevance, date, or geographic location and populates the entire dashboard with data from the resulting set of documents. The documents tab, discussed in Section 3, presents a subset of 50 documents in a list with the title, date of publication, overall sentiment, selected excerpt and source. As an alternative to the documents tab, users may choose to analyze the content at a sentence level using the second tab. This view displays a *concordance list* of quotations grouped by document and centered on the search term. Users can sort the results by source, date of publication, and the sentiment on both the document level and the sentence level. Clicking on a quotation activates a more detailed view of the document to which it belongs, displaying its title and the full sentences; a second click activates the full text mode, showing the document’s full text, keywords, and other annotations including source category, source location, target location, sentiment, and relevance. This concordance list provides a method for analysts to quickly scan the content and then drill down to examine a document in detail.

The *word tree* module (Fischl and Scharl, 2014, Scharl et al., 2014) presents the concordance list in a more intuitive manner, showing the different contexts in which fictional characters or topics are being discussed. Its graph-based display facilitates the rapid exploration of search results and conveys a better understanding of how language is being used surrounding a topic of interest. The *word tree* is based on the popular keyword-in-context technique (Wattenberg & Viégas, 2008) and adopts a symmetrical approach (Muralidharan, Hearst, & Fan, 2013). The root of the tree is the search term. The left part of the tree displays all sentence parts that occur before the search term (prefix tree), the right part those that follow the search term (suffix tree) - helping users to spot repetition in contextual phrases that precede or follow the search term. Visual cues include different font sizes to indicate the frequency of phrases, and connecting lines to highlight typical sentence structures. By hovering over a certain term or sub-tree, users can explore the lexical relations and track the individual connections between pre- and suffix sentence parts.

**Fig. 3:**
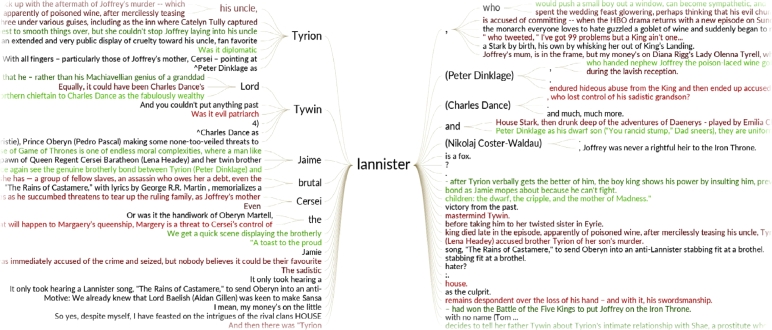
Word tree representation of search results for the query “lannister” in Anglo-American news media between March and April 2014.

Fig. 3 shows how the tree-like structure is built by (i) searching for the term “lannister”, (ii) grouping identical phrases containing the term into nodes – e.g., “Tyrion Lannister”, and (iii) creating additional sub-nodes once the sentences start to differ – e.g., “Tyrion Lannister (Peter Dinklage)”. This grouping together of equal phrases into a connected tree structure summarizes word usage within the selected source(s) in a given time interval.

### 4.2 Emotional context

The *radar chart* is a visual tool that goes beyond sentiment trend charts by profiling a topic (or entity) across several emotional categories. We selected emotional categories as defined in SenticNet 3, a semantic resource for concept-level opinion mining (Cambria et al., 2014). Because it provides knowledge on the three main levels ‘polarity’, ‘semantics’, and ‘sentics’, it caters to the needs of a state-of-the-art opinion mining toolkit, allowing for computation beyond mere keyword lookup or traditional machine learning. In particular, the ‘sentic’ level provides valuable insight into the four categories ‘pleasantness’, ‘sensitivity’, ‘aptitude’, and ‘attention’. Backed by Plutchik’s theoretical model on the nature of emotions (Plutchik, 2001), SenticNet aligns its 30,000 terms to these four dimensions. Weights indicate the strength of affiliation to the respective category, and one and the same term can be assigned to multiple categories.

#### 4.2.1 Visual representation of emotional categories

**Fig. 4:**
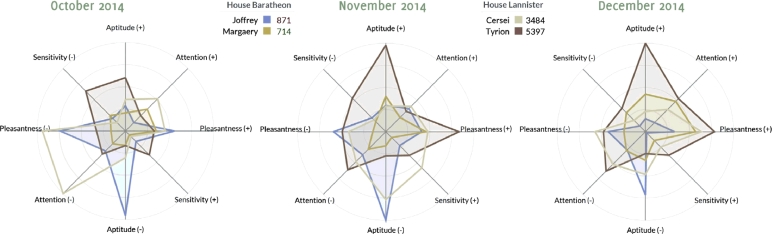
Series of radar charts showing media associations by SenticNet 3 category.

The *Westeros Sentinel* portrays how particular terms (e.g. entities such as actors or fictional characters) are perceived along these fours emotional categories. To capture these perceptions, the system has to derive two distinct values per search query, one positive and one negative for each of the emotional categories. During preprocessing, we store the 50 most positive terms and 50 most negative terms from each SenticNet category. Then, when users complete a search query, we calculate the number of documents that match both the search query as well as the 50 category terms. This step is repeated for each category, and for each positive and negative term list. Finally, we normalize these values using the total number of documents matching the search query alone. To compare entities, the multi-dimensional radar chart of Fig. 4 visualizes media perceptions by SenticNet categories. The example shows evolving associations with four fictional Game of Thrones characters – *Joffrey* and *Margaery Baratheon, Cersei* and *Tyrion Lannister* – based on news and social media coverage between October and December 2014.

The colored lines connecting the data points represent the selected topics. The radar chart supports ad-hoc data exploration and shows updates in real time. Since users can define topics on the fly, the comparison is not restricted to pre-defined characters or actors, but can be used to explore any topic of interest. This represents a holistic and real-time approach to visualizing affective knowledge in the underlying document sources, applicable to one or multiple targets without fragmenting the contextualized information space.

#### 4.2.2 Evaluation of knowledge extraction methods

The emotional categories shown in Fig. 4 were computed using association-based measures, which use significant phrase detection in conjunction with co-occurrence analysis based on a simple bag-of-words approach. The goal of this paper was not to advance and evaluate the underlying language processing methods as such, but to present a visual dashboard that demonstrates the usefulness of such measures for exploring domain-specific content repositories. While the evaluation of the dashboard itself is presented in Section 3.1, the performance of the underlying methods used to extract factual and affective knowledge (see Section 2.2) has already been reported in previous work:

• *Factual Knowledge:* The Recognyze named entity linking component uses linked data repositories to disambiguate and ground named entities. In contrast to machine learning approaches, Recognyze does not require annotated training data, but can be deployed for any domain or language as long as appropriate linked data resources are available. Depending on the used evaluation corpus, Recognyze yields a recall of 0.72 for identifying the most relevant organization in an article and an *F*-measure (hybrid metric to assess overall performance, balancing the typical trade-off between a method’s precision and recall) of up to 0.63 for named entity linking (Weichselbraun, Streiff, et al., 2014). Although the literature has reported higher accuracies for certain machine learning techniques, Recognyze is better suited for many real-world applications since it is not limited to a particular knowledge source and does not require annotated training corpora.
• *Affective Knowledge:* The sentiment analysis component (Weichselbraun, Streiff, et al., 2014) and its context-aware extension (Weichselbraun et al., 2013) provide the annotations required for the color coding of the visual methods presented in this paper. The contextualization process increased the observed average *F*-measure from 64.2 to 73.7 across all evaluation corpora. Broken down by category, the context-aware extension achieved an *F*_H_ (TripAdvisor hotel reviews) of 81 for positive and 72 for negative polarity, an *F*_P_ (Amazon product reviews) of 72 for positive and 69 for negative polarity, and an *F*_M_ (IMDB movie reviews) of 75 for positive and 73 for negative polarity (Weichselbraun et al., 2013). A follow-up evaluation showed that the observed *F*-measure improvement remains stable when classifying the reviews contained in the evaluation corpora, for example by product type or movie genre (Weichselbraun, Gindl, et al., 2014).

### 4.3 Relations among named entities

**Fig. 5:**
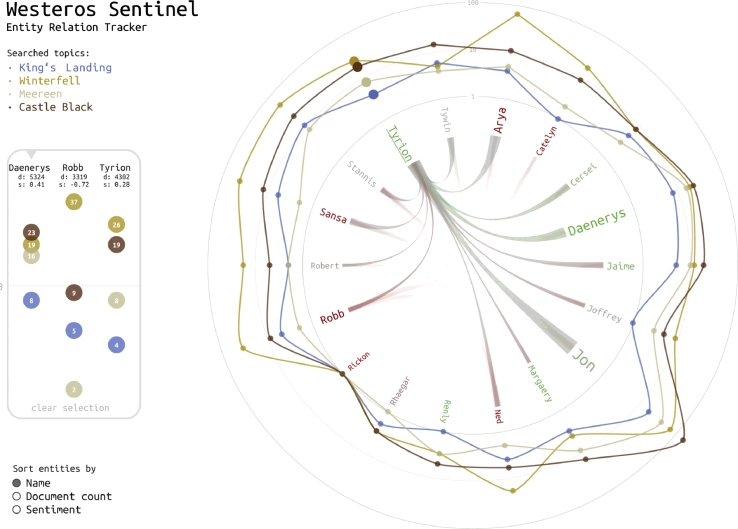
The *entity map* shows the sentiment toward Game of Thrones characters, and the relation among them based on co-occurrences in news and social media coverage.

The purpose of the *entity map* is to visualize co-occurrence patterns between named entities referenced in the selected corpora, and between these entities and user-defined search terms. In the case of the *Westeros Sentinel*, examples of entities include popular characters such as *Daenerys Targaryen* and *Tyrion Lannister,* or locations such as *King’s Landing, Winterfell* and *Meereen*. The entity map of Fig. 5 combines two different visualization tools: (i) a line chart with a radial imposition, and (ii) a radial convergence diagram.

• The *line chart* displays entity names along a circle – their font size relates to the number of documents that mention the entity, their color ranges from red to green depending on the average sentiment. The data points of each entity represent the number of co-occurrences between the entity and the user’s search terms, using a logarithmic scale. The system assigns a specific color to each search term (listed in the upper-left hand corner) and uses this color for the data points and connecting lines. Up to ten different search terms can be activated at any given time.
• The *radial convergence diagram* appears in the center of the graph. It displays relationships between entities using ribbons. The thickness of an arc represents the number of co-occurrences between an entity pair. On mouse-over, the opacity of arcs that connect the selected entity to other entities is increased.

Sorting functionality enables analysts to control how information is displayed. Clicking on a sorting category in the lower left corner causes the entities to be re-arranged (i) alphabetically by name, (ii), by the total number of documents which contain an instance of the entity’s name in descending order, and (iii) by the average sentiment of the documents containing the entity, from positive to negative. Both the line chart and the radial convergence diagram are being updated through smooth transitions. Hovering over an entity highlights the sector in the line chart, shows a tooltip with the top three keywords associated with this entity, and strengthens the corresponding arcs in the radial convergence diagram.

Additional interactions allow for more detailed comparisons. Clicking on an entity causes it to be displayed in a sidebar on the left-hand side, which displays the data points with the co-occurrence values and the entity information – i.e., name, document count (d), and sentiment (s). The logarithmic scale of the sidebar will adjust automatically to better accommodate the range of data values. The box contains the three most recently selected entities, which also remain highlighted in the graph.

## 5 Conclusion and outlook

This paper described the *Westeros Sentinel*, a Web intelligence application to analyze news and social media coverage about Game of Thrones, the American television series based on George R.R. Martin’s series of fantasy novels. The system’s capabilities to aggregate and analyze public discourse were presented using data collected during the television series’ fourth season that premiered on 2 April 2014. The system extracts information from the Web sites of Anglo-American news media and four social media platforms – Twitter, Facebook, Google+ and YouTube. A real-time dashboard offers various visualizations to track how often characters are being mentioned by journalists and viewers, and to identify concepts that are being associated with the unfolding storyline and each new episode. Sentiment annotations and affective knowledge according to the SenticNet 3 categories *pleasantness, aptitude, attention*, and *sensitivity* (Cambria et al., 2014) shed light on the perception of actors and new plot elements.

The *Westeros Sentinel* is built on top of the webLyzard Web intelligence platform (www.weblyzard.com), which goes beyond traditional audience measures. It helps establish connections between trends and entities in real time, using data mining in conjunction with information visualization to aggregate and present content originating from multiple online sources. Uncovering patterns and trends in these sources is helpful for engaging audiences, optimizing communication strategies, and increasing the effectiveness of decision making processes. Data integration and contextualization services not only facilitate accessing various sources of digital content, but also transform unstructured repositories of public discourse into actionable knowledge. This paper has presented technologies to build such services, which can provide significant value for a wide range of organizations including enterprises, non-government entities, news media outlets, science agencies, and policy makers.

Future work will extend the presented knowledge extraction framework with a dependency parsing component for extracting complex features. Interactive visualizations showing a subset of these features will help evaluators not only to assess the overall sentiment of a document, but also identify opinion holders and opinion targets referenced in this document. In the context of the *Westeros Sentinel*, this will help to better distinguish discussions about the storyline of an episode from media critiques or assessments of a specific actor’s performance.

## Figures and Tables

**Table 1:** Selected characters from Game of Thrones, including the corresponding sets of regular expressions used to filter and pre-process the content streams.

Character	Input Filter|Regular Expression
Arya Stark	(Princess of Winterfell|Arya (Horseface|Stark|Underfoot)) |Arry|Lumpy(face|head)|Weasel|Nymeria|Salty|Cat of the Canals|The (Blind|Ugly Little) Girl|Mercedene|Mery)
Sansa Stark	(Lady of Winterfell|Little (Dove|Bird)|Sansa (Stark|Lannister))
Joffrey Baratheon	(Joffrey (I.?)?Baratheon|King Joffrey)
Tyrion Lannister	(Tyrion Lannister|The Imp|Halfman|The Little Lion|Demon Monkey)
Daenerys Targaryen	(Daenerys (Targaryen|Stormborn)|Dany|Mother of Dragons|Mhysa|The (Queen Across the Sea|Silver Queen|Beggar Queen))
